# Understanding Perceptions, Knowledge and Implementation Barriers of Enhanced Recovery After Surgery Among Surgeons and Anesthesiologists

**DOI:** 10.7759/cureus.79595

**Published:** 2025-02-24

**Authors:** Abdul Haseeb, Muhammad Zeb, Khola Darain, Haris Ali, Rafia Ahmad, Javeria Shah, Hira Bakhtiar Khan, Muhammad Raheel, Diyan Muhammad

**Affiliations:** 1 Urology, Hayatabad Medical Complex Peshawar, Peshawar, PAK; 2 General Surgery, Hayatabad Medical Complex Peshawar, Peshawar, PAK; 3 General Surgery, Khyber Teaching Hospital, Medical Teaching Institution (MTI), Peshawar, PAK; 4 General Surgery, Lady Reading Hospital, Medical Teaching Institution (MTI), Peshawar, PAK; 5 Urology, Leicester General Hospital, University Hospitals of Leicester (UHL) Trust, Leicester, GBR

**Keywords:** : enhanced recovery after surgery (eras), implementation barriers, knowledge, perception, surgeons

## Abstract

Objective

The present study aimed to evaluate the knowledge, perceptions, and barriers associated with Enhanced Recovery After Surgery (ERAS) implementation among perioperative clinicians.

Methods

This cross-sectional study included responses from 214 perioperative clinicians, comprising surgeons and anesthesiologists involved in abdominal surgeries, from tertiary care hospitals in Khyber-Pakhtunkhwa, Pakistan. A structured questionnaire collected data on participants’ knowledge of ERAS protocols, perceived barriers to implementation, and learning preferences. Responses were analyzed using descriptive and inferential statistics, with significance set at p<0.05.

Results

This study included 214 perioperative clinicians, predominantly residents (91.6%, n=196), from surgery (90.2%, n=193) and anesthesiology (9.8%, n=21) departments. Knowledge about ERAS protocols was limited, with 89 (41.6%) of respondents stating they knew nothing and 97 (45.3%) reporting very little or some familiarity. Perceived barriers included lack of institutional support, time constraints, and insufficient research. Most participants, 145 (67.8%), supported integrating ERAS education into formal training, with 92 (42.9%) favoring seminars or lectures and 79 (36.9%) preferring journal articles for learning. Perceptions of ERAS importance were generally positive, but significant differences were noted regarding hospital administration support (p=0.013).

Conclusion

This study identifies significant gaps in ERAS knowledge among perioperative clinicians, particularly among residents, and highlights perceived logistical barriers to its implementation. However, the findings are limited by the underrepresentation of consultants and anesthesiologists, who are key drivers of ERAS programs. The findings highlight the need for targeted educational interventions, stronger institutional support, and multidisciplinary collaboration to improve ERAS adoption.

## Introduction

Enhanced Recovery After Surgery (ERAS) protocols are structured, multidisciplinary perioperative care pathways originally developed to optimize recovery following abdominal surgeries, particularly colorectal procedures. Over time, their principles have been adapted to various surgical specialties to enhance patient outcomes and reduce perioperative complications. By preserving preoperative organ function and mitigating the physiological stress response associated with surgery, ERAS protocols aim to enhance patient outcomes and reduce the physical burden of surgical interventions. These evidence-based protocols encompass key elements such as patient counseling and education, preoperative optimization, avoidance of prolonged fasting, oral carbohydrate loading, opioid-sparing analgesia, regional anesthesia, prophylaxis for postoperative nausea and vomiting (PONV), appropriate antibiotic administration, and early mobilization. Collectively, these measures improve patient care, reduce complications, and shorten hospital stays [1-3].

Despite the well-documented benefits of ERAS protocols, their implementation in clinical practice remains challenging. Numerous studies have identified critical barriers, including skepticism regarding the real-world applicability of ERAS and low expectations concerning its impact on patient outcomes. While overall awareness of the formal ERAS bundle may be limited, it is possible that certain ERAS-aligned practices - such as minimizing preoperative fasting, reducing opioid use, early mobilization, and avoiding routine drains or nasogastric tubes - are already being followed in many centers, albeit without explicit ERAS labeling [4].

A survey conducted in Australia and New Zealand reported that only 37% of specialist colorectal surgeons routinely implemented ERAS pathways in patient care. The study identified a lack of institutional support (39%) as the most significant barrier, followed by limited interest from co-specialty personnel (33%). Although 68.4% of respondents were at least “moderately” familiar with ERAS, they cited insufficient administrative backing and inadequate access to educational resources as major hurdles to successful implementation [5]. Similarly, a multicenter survey from institutions in Switzerland and Sweden found that while healthcare providers acknowledged ERAS protocols’ potential to reduce complications, improve patient satisfaction, and shorten hospital stays, they faced persistent barriers such as time constraints, logistical challenges, and reluctance to adopt new practices [6]. Furthermore, studies have reported a lack of understanding regarding the optimal methods for educating and engaging resident physicians in ERAS-based patient care, further complicating implementation efforts [7].

The present study aims to evaluate the knowledge, perceptions, and barriers associated with ERAS implementation among perioperative clinicians. By exploring these factors, we seek to identify areas for improvement and contribute to the establishment of quality evaluation systems for ERAS protocols. The findings of this study will provide critical insights into the challenges of implementing ERAS programs, guiding future initiatives to enhance adoption and improve perioperative care, particularly within low- and middle-income countries (LMICs).

## Materials and methods

This cross-sectional study surveyed perioperative clinicians from general surgery and anesthesiology departments involved in abdominal surgeries at tertiary care hospitals in Peshawar, Pakistan, from 1st December 2023 to 31st December 2023. The study aimed to assess the knowledge, perceptions, and barriers to the implementation of ERAS protocols among perioperative clinicians. The target population included faculty general surgeons, anesthesiologists, and postgraduate trainees actively involved in perioperative abdominal surgeries. A total of 214 participants were recruited through convenience sampling, including both residents and consultants from the general surgery and anesthesiology departments.

The required sample size was determined using Cochran’s formula for cross-sectional studies, assuming a 50% prevalence of ERAS familiarity due to the lack of prior regional data, a 95% confidence level, and a 7% margin of error. The calculated sample size was 196, which was increased by 10% to account for non-response, resulting in a target of 216 participants. A total of 250 questionnaires were distributed, and 214 completed responses were received, yielding a response rate of 85.6%.

The survey instrument was adapted from a previously conducted study by Beal EW et al. [8] and was designed to assess multiple dimensions of ERAS implementation. These included familiarity with ERAS protocols, perceived value, barriers to adoption, and preferred learning methods. The questionnaire collected demographic data such as age, gender, specialty, designation (Consultant or Resident), postgraduate year (PGY) of residents, and years of clinical experience for consultants. Additionally, participants were asked whether they had ever been involved in the care of a patient following an ERAS protocol. Responses were recorded on a five-point Likert scale to assess the level of agreement or disagreement with various statements regarding ERAS (See Appendix).

The study focused on several key parameters, including knowledge and awareness (familiarity with ERAS principles, purpose, and components), perceptions and attitudes (views on ERAS effectiveness, perceived benefits, and institutional support), barriers to implementation (identified obstacles in the application of ERAS protocols), preferred learning methods (workshops, online courses, mentorship, or clinical exposure), and clinical experience with ERAS (prior involvement in patient care under ERAS protocols).

Sample selection

The study included perioperative clinicians, comprising general surgeons and anesthesiologists especially involved in abdominal surgeries, from tertiary care hospitals in Khyber-Pakhtunkhwa, Pakistan. Participants who were non-clinical personnel, clinicians not directly involved in perioperative care, those who provided incomplete or inconsistent responses, or those who refused to provide informed consent were excluded from the study.

Ethical approval was obtained from the Institutional Review Board (Reference No: HMC-QAD-F-1664), and informed consent was secured from all participants before data collection. To ensure confidentiality, responses were collected anonymously, and no identifying information was recorded.

Data analysis

Data were collected using Google Forms and analyzed using SPSS version 23 (IBM Corp., Armonk, NY, USA). Descriptive statistics were used to summarize categorical variables, such as demographics, knowledge levels, and perceptions, through frequencies and percentages. Cross-tabulations were performed to compare responses between residents and consultants. Chi-square tests were used to determine associations between designation and various aspects of ERAS knowledge and implementation, including ERAS knowledge levels, perceptions of ERAS importance, views on hospital administration and colleague support, beliefs about patient care improvement, perceptions of time investment, ERAS protocol participation, understanding of ERAS purpose, preferred learning methods, and views on ERAS implementation scope. A p-value <0.05 was considered statistically significant, and all statistical tests were two-tailed. 

## Results

This study included 214 perioperative clinicians with varying specialties, genders, designations, and levels of experience. The majority of participants were from the field of surgery, n=193 (90.2%), while only n=21 (9.8%) were anesthesiologists. Male participants predominated n=157 (73.4%), with females comprising n=57 (26.6%). Most respondents were residents, n=196 (91.5%), while consultants accounted for only n=18 (8.4%). Consultants’ years of experience varied, with most having less than five years of practice, n=10 (55.6%), followed by five to 10 years, n=3 (16.7%), 10-15 years, n=3 (16.7%), and 15-20 years, n=2 (11.1%) (summarized in Table 1).

**Table 1:** Summary of Demographic Variables PGY: Postgraduate Year

Demographic Variables	Groups	Number (n)	Frequency (%)
Specialty	Surgery	193	90.2
	Anesthesia	21	9.8
	Total	214	100.0
Gender	Male	157	73.4
	Female	57	26.6
	Total	214	100.0
Designation	Resident	196	91.6
	Consultant	18	8.4
	Total	214	100.0
Post Graduate Year (PGY)	Year 1	44	20.6
	Year 2	54	25.2
	Year 3	43	20.1
	Year 4	46	21.5
	Year 5	9	4.2
	Total	196	91.6
Consultant's Years of Experience	<5 yrs	10	55.6
	5-10yrs	3	16.7
	10-15 yrs	3	16.7
	15-20yrs	2	11.1
	Total	18	100.0

Comparison of ERAS knowledge and institutional support among consultants and residents

Comparison of designation with perceptions about ERAS is summarized in Table 2. The findings indicate that knowledge about ERAS protocols is limited among perioperative clinicians, with n=89 (41.6%) participants, including n=79 (40.3%) of residents and n=10 (5.1%) of consultants, reporting no knowledge of ERAS. A smaller proportion, n=51 (23.8%), had very little knowledge, while n=46 (21.5%) reported some familiarity. Only n=23 (10.7%) had significant knowledge, and n=5 (2.3%) stated they knew everything about ERAS.

**Table 2:** Comparison of ERAS Knowledge and Institutional Support among Consultants and Residents ERAS: Enhanced Recovery After Surgery P-value of <0.05 was considered significant. Significant P-value is highlighted in bold letters in the table

PERCEPTION		Designation		Total	P-value
		Resident (n=196)	Consultant (n=18)		
I know __________ about enhanced recovery after surgery(ERAS) protocols	Nothing	79 (40.3%)	10 (55.6%)	89 (41.6%)	0.680
	Very Little	48 (24.5%)	3 (16.7%)	51 (23.8%)	
	Some	42 (21.4%)	4 (22.2%)	46 (21.5%)	
	A Lot	22 (11.2%)	1 (5.6%)	23 (10.7%)	
	Everything	5(2.3%)	0 (0.0%)	5(2.3%)	
I believe enhanced recovery protocols are important for patient care	Strongly Disagree	67 (34.2%)	3 (16.7%)	70 (32.7%)	0.502
	Disagree	15 (7.7%)	3 (16.7%)	18 (8.4%)	
	Neutral	28 (14.3%)	3 (16.7%)	31 (14.5%)	
	Agree	48 (24.5%)	5 (27.8%)	53 (24.8%)	
	Strongly Agree	38 (19.4%)	4 (22.2%)	42 (19.6%)	
I believe the hospital administration thinks enhanced recovery protocols are important for patient care	Strongly Disagree	44 (22.4%)	1 (5.6%)	45 (21.0%)	0.013*
	Disagree	6 (3.1%)	0 (0.0%)	6 (2.8%)	
	Neutral	39 (19.9%)	10 (55.6%)	49 (22.9%)	
	Agree	73 (37.2%)	5 (27.8%)	78 (36.4%)	
	Strongly Agree	34 (17.3%)	2 (11.1%)	36 (16.8%)	
I believe my colleagues think enhanced recovery is important for patient care	Strongly Disagree	31 (15.8%)	3 (16.7%)	34 (15.9%)	0.869
	Neutral	7 (3.6%)	0 (0.0%)	7 (3.3%)	
	Agree	69 (35.2%)	7 (38.9%)	76 (35.5%)	
	Strongly Agree	89 (45.4%)	8 (44.4%)	97 (45.3%)	
I believe that my patients have/will have improved care when they are involved in enhanced recovery protocols	Strong Disagree	41 (20.9%)	7 (38.9%)	48 (22.4%)	0.473
	Disagree	1 (0.5%)	0 (0.0%)	1 (0.5%)	
	Neutral	22 (11.2%)	1 (5.6%)	23 (10.7%)	
	Agree	68 (34.7%)	6 (33.3%)	74 (34.6%)	
	Strongly Agree	64 (32.7%)	4 (22.2%)	68 (31.8%)	
I believe the enhanced recovery protocols is a reasonable investment of my time	Strongly Disagree	45 (23.0%)	3 (16.7%)	48 (22.4%)	0.637
	Neutral	27 (13.8%)	1 (5.6%)	28 (13.1%)	
	Agree	73 (37.2%)	8 (44.4%)	81 (37.9%)	
	Strongly Agree	51 (26.0%)	6 (33.3%)	57 (26.6%)	
I have participated in the care of a patient in an ERAS protocol	Yes	120 (61.2%)	11 (61.1%)	131 (61.2%)	0.590
	No	76 (38.8%)	7 (38.9%)	83 (38.8%)	

Regarding the importance of ERAS protocols in patient care, opinions were mixed. A considerable n=70 (32.7%) participants strongly disagreed with their importance, with n=67 (34.2%) residents and n=3 (16.7%) consultants sharing this view. Meanwhile, n=53 (24.8%) agreed, and 42 (19.6%) strongly agreed. The perception of hospital administration’s support for ERAS varied significantly (p=0.013), with n=45 (21.0%) respondents strongly disagreeing, including n=44 (22.4%) residents and n=1 (5.6%) consultant. A total of n=78 (36.4%) agreed that their administration supported ERAS, while n=36 (16.8%) strongly agreed.

Colleague support for ERAS was perceived positively, as n=97 (45.3%) strongly agreed that their peers valued ERAS, and n=76 (35.5%) agreed. However, n=34 (15.9%) strongly disagreed, demonstrating some level of skepticism among the respondents. Similarly, the belief that ERAS improves patient care was acknowledged by n=74 (34.6%) agreeing and n=68 (31.8%) strongly agreeing, while n=48 (22.4%) strongly disagreed.

Regarding the investment of time in ERAS protocols, n=81 (37.9%) agreed, and n=57 (26.6%) strongly agreed that it was worthwhile, but n=48 (22.4%) strongly disagreed. Participation in ERAS-based patient care was reported by n=131 (61.2%) of the respondents, with similar proportions among residents n=120 (61.2%) and consultants n=11 (61.1%), while n=83 (38.8%) had not participated.

Comparison of learning preferences and barriers to ERAS implementation among consultants and residents

When asked about the purpose of ERAS protocols, n=92 (42.9%) believed they address patient expectations preoperatively to improve satisfaction, while n=57 (26.6%) highlighted attenuating surgical stress. A smaller percentage, n=40 (18.7%), noted improving hospital efficiency, and n=25 (11.7%) selected “all of the above.” There were no significant differences between groups (p=0.805). Participants were most interested in minimizing perioperative complications n=66 (30.8%) and improving efficiency n=64 (29.9%), with others focusing on fluid management n=44 (20.6%) and multimodal pain management n=23 (10.7%). Only n=17 (8.0%) expressed interest in all areas (p=0.857). The preferred method for learning ERAS was seminars or lectures n=92 (42.9%), followed by journal articles n=79 (36.9%), and direct participation in institutional protocols n=43 (20.1%) (p=0.680). Most participants n=145 (67.8%) supported incorporating ERAS education into formal training, with no significant differences between groups (p=0.349). The most cited barriers to ERAS knowledge were a lack of patient interest n=76 (35.5%), insufficient institutional information n=51 (23.8%), and lack of research n=52 (24.3%). Time constraints n=15 (7.0%) and lack of surgeon interest n=20 (9.3%) were less frequently noted (p=0.490). Finally, n=169 (79.0%) of respondents supported broad implementation of ERAS, while n=45 (21.0%) believed it should be focused on specific patient populations, and no significant differences were observed between residents and consultants' responses (p=0.225) (summarized in Table 3).

**Table 3:** Comparison of Learning Preferences and Barriers to ERAS Implementation among Consultants and Residents ERAS: Enhanced Recovery After Surgery P value of <0.05 was considered significant

Broad Questions	Sub questions	Residents (n=196)	Consultants (n=18)	Total	P-value
Enhanced Recovery After Surgery (ERAS) protocols are primarily designed to:	Attenuate surgical stress	52 (24.3%)	5 (2.3%)	57 (26.6%)	0.805
	Improve efficiency and financial return	36 (16.8%)	4 (1.9%)	40 (18.7%)	
	Address patient expectations	86 (40.2%)	6 (2.8%)	92 (43.0%)	
	All of the above	22 (10.3%)	3 (1.4%)	25 (11.7%)	
Interested in learning more about	Fluid Management	39 (18.2%)	5 (2.3%)	44 (20.6%)	0.857
	Multimodal Pain Management	22 (10.3%)	1 (0.5%)	23 (10.7%)	
	Minimizing Perioperative Complications	61 (28.5%)	5 (2.3%)	66 (30.8%)	
	Improving Perioperative Efficiency	59 (27.6%)	5 (2.3%)	64 (29.9%)	
	All of the above	15 (7.0%)	2 (0.9%)	17 (8.0%)	
Preferred method to learn about ERAS	Institutional participation	39 (18.2%)	4 (1.9%)	43 (20.1%)	0.680
	Journal articles	71 (33.2%)	8 (3.7%)	79 (36.9%)	
	Seminars or lectures	86 (40.2%)	6 (2.8%)	92 (43.0%)	
Formal education on ERAS in training	Yes	134 (62.6%)	11 (5.1%)	145 (67.8%)	0.349
	No	62 (29.0%)	7 (3.3%)	69 (32.2%)	
Barriers to gaining knowledge	Lack of Research	46 (21.5%)	6 (2.8%)	52 (24.3%)	0.490
	Lack of Time	14 (6.5%)	1 (0.5%)	15 (7.0%)	
	Lack of Institutional Information	48 (22.4%)	3 (1.4%)	51 (23.8%)	
	Lack of Patient Interest	68 (31.8%)	8 (3.7%)	76 (35.5%)	
	Lack of Surgeon Interest	20 (9.3%)	0 (0.0%)	20 (9.3%)	
ERAS protocols should be:	Implemented Broadly	153 (71.5%)	16 (7.5%)	169 (79.0%)	0.225
	Focused on Specific Populations	43 (20.1%)	2 (0.9%)	45 (21.0%)	

## Discussion

This study evaluated perioperative clinicians' perceptions, knowledge, implementation challenges, and learning preferences regarding ERAS protocols. Despite the well-documented benefits of ERAS in improving patient outcomes, reducing complications, and shortening hospital stays, significant knowledge and perception gaps persist among healthcare professionals.

Survey results revealed that nearly half of the participants, predominantly residents, were unaware of ERAS protocols, with most rating their knowledge as 'very little' or 'some'. Specifically, n=51 (23.8%) participants reported knowing "very little," while n=46 (21.5%) indicated knowing "some." A smaller fraction demonstrated a comprehensive understanding, with n=23 (10.7%) stating they knew "a lot" and only n=5 (2.3%) claiming full knowledge. These findings align with Beal et al. and Ahmad H et al., who reported significant gaps in ERAS knowledge among clinicians [8,9]. However, it is important to note that our study predominantly reflects the perspectives of residents, who are not formally trained in ERAS protocols, unlike the more diverse clinician populations in the cited studies.

Most respondents, predominantly residents, acknowledged ERAS's importance in enhancing patient care. However, perceptions of administrative and institutional support varied. The perspectives of consultants and anesthesiologists, who were underrepresented in the study. While n=114 (53.2%) participants agreed that hospital administration values ERAS, n=51 (23.8%) disagreed, indicating inconsistent institutional commitment. This variability is consistent with Cohen and Gooberman-Hill’s findings, which underscore the role of multidisciplinary team (MDT) communication and collaboration in successfully integrating ERAS [10].

The majority of participants believed their colleagues valued ERAS. This optimism reflects a positive perception of ERAS among peers. However, Pearsall et al. noted that differing expectations across MDTs and incorrect assumptions about colleagues’ knowledge can create uncertainty, potentially hindering implementation. These findings highlight the need for clear communication and shared goals within perioperative teams to foster a supportive ERAS culture [11].

Key barriers to ERAS implementation identified in this study, primarily from the perspective of residents, included insufficient research, time constraints, limited institutional support, and a lack of interest from both patients and surgeons. Similarly, Herbert G et al., emphasized that resource limitations, high staff turnover, and time constraints impede ERAS adoption [12]. Addressing these barriers requires targeted efforts to enhance institutional resources, promote staff education, and streamline implementation processes.

Regarding learning preferences, n=92 (42.9%) participants favored seminars or lectures, n=79 (36.9%) preferred journal articles or textbooks, and n=43 (20.1%) valued direct participation in institutional protocols. The majority of participants agreed that formal ERAS education should be integrated into medical training, a finding consistent with existing literature advocating for ERAS inclusion in residency curricula to improve awareness and implementation [8].

The findings of this study primarily reflect the perspectives of residents, who constitute the majority of the sample. While residents are actively involved in perioperative care, their limited exposure to ERAS protocols in the training curriculum may have influenced the results. The underrepresentation of consultants and anesthesiologists, who are typically key drivers of ERAS programs, is a significant limitation. Future research should prioritize engaging these groups to better understand their perspectives and roles in ERAS implementation.

Limitations

The study has several limitations. First, the overrepresentation of residents and underrepresentation of consultants and anesthesiologists may limit the generalizability of the findings. Consultants and anesthesiologists are typically key drivers of ERAS programs, and their limited participation is a notable gap in our study. Second, ERAS protocols are not formally included in the training curriculum for residents in our region, which likely contributed to the limited knowledge observed in this group. Future studies should focus on engaging consultants and anesthesiologists to provide a more comprehensive understanding of ERAS implementation barriers.

## Conclusions

This study identifies significant gaps in ERAS knowledge among perioperative clinicians, particularly among residents, and highlights perceived logistical barriers to its implementation. However, the findings are limited by the underrepresentation of consultants and anesthesiologists, who are key drivers of ERAS programs. The findings highlight the need for targeted educational interventions, stronger institutional support, and multidisciplinary collaboration to improve ERAS adoption.

## Appendices

**Table 4:** Survey Questionnaire ERAS: Enhanced Recovery After Surgery Survey adapted from [8]

Section	Field/Question	Options/Response Choices
Consent	Consent	________________
I. Demographics and Experience		
	1. Department	□ Surgery □ Anesthesia
	2. Role	□ Consultant □ Resident
	a. If Resident, PGY-Year	□ 1 □ 2 □ 3 □ 4 □ 5 □ 6 □ 7 □ 8 □ 9 □ 10
	b. If Consultant, Years in Practice	□ <5 □ 5-10 □ 11-15 □ 16-20 □ 21-25 □ >25
	3. Gender	□ Male □ Female
	4. Age	________________
	5. Primary Hospital	□ HMC □ KTH □ LRH □ RMI □ NWGH □ ATH □ MMC □ CMH □ STH □ QHAMC □ Other: ________________
	6. ERAS Experience	□ Yes □ No
II. Perceptions About ERAS		
	8. I know _____ about ERAS protocols	□ Nothing □ Very little □ Some □ A lot □ Everything
	9. ERAS protocols are important for patient care	□ Strongly Disagree □ Disagree □ Neutral □ Agree □ Strongly Agree
	10. Hospital administration thinks ERAS protocols are important	□ Strongly Disagree □ Disagree □ Neutral □ Agree □ Strongly Agree
	11. My colleagues think ERAS protocols are important	□ Strongly Disagree □ Disagree □ Neutral □ Agree □ Strongly Agree
	12. Patients will have improved care with ERAS protocols	□ Strongly Disagree □ Disagree □ Neutral □ Agree □ Strongly Agree
	13. ERAS protocols are a reasonable investment of my time	□ Strongly Disagree □ Disagree □ Neutral □ Agree □ Strongly Agree
	14. ERAS protocols improve institutional financial efficiency	□ Strongly Disagree □ Disagree □ Neutral □ Agree □ Strongly Agree
III. Knowledge Assessment		
	1. Enhanced recovery protocols are primarily designed to:	□ a. Attenuate the patient's response to surgical stress to improve length of stay and reduce postoperative complications and mortality □ b. Improve the efficiency of the hospital and lead to improved financial return in a diagnosis-related group payment system □ c. Address patient expectations preoperatively to lead to improved patient satisfaction □ d. All of the Above
IV. Future Planning		
	1. Enhanced recovery protocols should be:	□ a. Implemented broadly □ b. Focused on specific patient populations
	2. I am most interested in learning more about the following elements of ERAS:	Fluid management Multimodal pain management Minimizing perioperative complications Improving perioperative efficiency
	3. My ideal method to learn about ERAS is:	□ a. Direct participation in institutional protocols □ b. Reviewing Journal articles or text books □ c. Seminars or lectures on the topic from national leaders □ d. Seminars or lectures on the topic from local leaders
	4. I think formal education about ERAS should be part of training for:	Surgeons: □ Yes □ No Anesthesiologist: □ Yes □ No
	5. I think barriers to gaining knowledge about ERAS include:	□ a. Lack of research □ b. Lack of time □ c. Lack of information provided by my employer □ d. Lack of interest from patients □ e. Lack of interest from providers
